# Molecular characterization and the mutation pattern of SARS-CoV-2 during first and second wave outbreaks in Hiroshima, Japan

**DOI:** 10.1371/journal.pone.0246383

**Published:** 2021-02-05

**Authors:** Ko Ko, Shintaro Nagashima, Bunthen E., Serge Ouoba, Tomoyuki Akita, Aya Sugiyama, Masayuki Ohisa, Takemasa Sakaguchi, Hidetoshi Tahara, Hiroki Ohge, Hideki Ohdan, Tatsuhiko Kubo, Eisaku Kishita, Masao Kuwabara, Kazuaki Takahashi, Junko Tanaka

**Affiliations:** 1 Department of Epidemiology, Infectious Disease Control and Prevention, Graduate School of Biomedical and Health Sciences, Hiroshima University, Hiroshima, Japan; 2 Payment Certification Agency, Ministry of Health, Phnom Penh, Cambodia; 3 Unité de Recherche Clinique de Nanoro (URCN), Nanoro, Burkina Faso; 4 Department of Virology, Graduate School of Biomedical and Health Sciences, Hiroshima University, Hiroshima, Japan; 5 Department of Cellular and Molecular Biology, Graduate School of Biomedical and Health Sciences, Hiroshima University, Hiroshima, Japan; 6 Department of Infectious Diseases, Hiroshima University Hospital, Hiroshima, Japan; 7 Department of Gastroenterological and Transplant Surgery, Graduate School of Biomedical and Health Sciences, Hiroshima University, Hiroshima, Japan; 8 Department of Public Health and Health Policy, Graduate School of Biomedical and Health Sciences, Hiroshima University, Hiroshima, Japan; 9 Hiroshima Prefecture Health and Welfare Bureau, Hiroshima, Japan; 10 Hiroshima Prefectural Center for Disease Control and Prevention, Hiroshima, Japan; University of Hong Kong, HONG KONG

## Abstract

**Background:**

In this study, we performed molecular characterization of SARS-CoV-2 strains in Hiroshima and its mutation pattern between the first and second waves of the outbreak.

**Method:**

A total of 55 nasal swab samples from the first wave in Hiroshima and 13 from the second wave were examined quantitatively by RT-qPCR and qualitatively by nested PCR using specific primers. Four samples from each wave underwent next-generation sequencing and phylogenetic tree analysis including controls and all sequences retrieved in Japan from GISAID and GenBank. Subsequently, mutations were examined.

**Results:**

Viral load ranged 7.85 × 10^1^−1.42 × 10^8^ copies/ml. Of 68 samples, one was Asian type-O, 65 were European type-GR, and 2 were undetectable. Phylogenetic tree analysis indicated that Japan was infected with various Asian strains (L, S, V, O) from January through April. By second week of March, European strains (G, GH, GR) had appeared, and GR strains became predominant after mid-March. The first case in Hiroshima was classified as Asian strain O, and the rest were GR strains. Then, second wave of GR strains appeared independently with 11–15 base mutations. Comparing the first- and second-wave GR strains, mutation rate was 1.17–1.36 × 10^−3^ base substitutions per site per year; in addition, amino acid changes occurred at S1361P and P3371S in ORF1a, A314V in ORF1b, and P151L in N. All seven GR strains were D614G variants with R202K and G203R mutations in N. A single-nucleotide insertion in *ORF8* that causes a defect in ORF8 protein was found in one isolate (S66) from the second wave.

**Conclusion:**

Our findings reveal the evolutionary hierarchy of SARS-CoV-2 in Japan. The predominant D614G variants and a new form of *ORF8* deletion in Hiroshima provide the clue for role of viral factor in local outbreaks of SARS-CoV-2.

## Background

Novel coronavirus, formerly named 2019-nCoV and later officially termed as SARS-CoV-2 by the International Committee on Taxonomy of Viruses (ICTV), is a member of genus *Betacoronavirus*, which also includes SARS-CoV-1 and MERS-CoV [1]. The novel coronavirus is a positive-sense single-strand RNA genome of approximately 30 kb, the largest genome among RNA viruses [2].

SARS-CoV-2 causes a lower respiratory tract infection that progresses to severe acute respiratory syndrome and multi-organ failure [3, 4]. More than 1 million people have lost their lives as a result of infection by this virus. After detection of SARS-CoV-2 in Wuhan, China in late December 2019, approximately 33 million confirmed cases and 996,309 deaths had been reported worldwide as of September 27, 2020 [5]. The World Health Organization (WHO) declared this infection as a pandemic on March 11, 2020 [6].

As of August 3, 2020, a total of 40,244 confirmed cases and 1,018 deaths had been reported in Japan [7]. The incidence rate differs across prefectures, ranging from 181.51 per 100,000 people in Tokyo to 2.81 per 100,000 people in Aomori prefecture [7]. The case-fatality ratio also differs between prefectures, ranging from 0% in Iwate and Akita prefectures to 1.48% in Tokyo.

The first wave of corona outbreak in Japan occurred between the last week of March and the first week of May 2020, with a peak of 701 newly confirmed cases per day. The second wave of the outbreak has been ongoing since the last week of June 2020, with a peak of 1,762 newly confirmed cases per day [7].

Hiroshima Prefecture, located in the southwest of Japan’s main island, had an estimated population of 1.1 million in 2019 [8]. The first wave of SARS-CoV-2 outbreak in Hiroshima, which occurred between the last week of March and the first week of May, affected 162 people. The second wave, from the last week of June to the first week of August 2020, affected 204 people [9]. Overall, as of August 3, 2020, Hiroshima has had a total of 366 confirmed cases with three deaths. Although confirmed cases are reported daily in Hiroshima, there is no detailed study on the molecular epidemiology of SARS-CoV-2 circulating in the prefecture.

In this study, we performed a molecular characterization of SARS-CoV-2 strains found in Hiroshima and compared mutations of these strains between the first and second waves of the outbreak.

## Materials and methods

### Subjects and their clinical data

55 nasal swab samples from confirmed cases of SARS-CoV-2 during the first wave and 13 samples from the second wave of outbreak were provided from Hiroshima Prefecture Research Institute after being used by government laboratory. The average age of patients in first wave was 62.2±23.9 years old whilst that from second wave was 33.1±14.9 years old. The proportion of male patients were 32.7% and 69.2% in first and second wave respectively. The anonymized clinical data were collected from the respective hospital. This information includes age, sex, date of onset, admission date, discharge date, symptoms, outcomes and the contact history.

### Nucleic acid extraction and quantitative measurement of SARS-CoV-2

Nasal swab samples (50 μl) were subjected to nucleic acid extraction using SMITEST EX-R&D (MBL, USA). The final pellet was diluted with 25 μl of RNase inhibitor–based water (Thermo Fisher Scientific, USA). For each sample, viral load was quantified by real-time reverse transcriptase polymerase chain reaction (RT-qPCR) (Thermo Fisher Scientific) using the nucleocapsid (N) gene-specific primers NIID_2019-n-CoV-N-F2 (targeting nt29,125–nt29,144) and NIID_2019-n-CoV-N-R2 (targeting nt29,299–nt29,280) and probe NIID_2019-n-CoV-N-P2 (nt29,222–nt29,241) (Eurofins Genomics, Japan).

### Classification of GISAID clade

To classify the GISAID clade, the partial genomes of SARS-CoV-2 were amplified from all samples by polymerase chain reaction (PCR) with 7 sets of primers as shown in Table 1A. The first round of PCR was done by Prime Script One-Step RT- PCR kit Ver.2 (Takara Bio Inc., Shiga, Japan) for 30 minutes at 50°C and 1 minute at 94°C. Then, the samples were exposed to 40 cycles each of 30 seconds at 94°C, 30 seconds at 55°C, 1 minute at 72°C and final elongation for 7 minutes at 72°C. Then, the second round of PCR was done by TaKaRa Ex Taq® Hot Start version (Takara Bio Inc., Shiga, Japan) to 30 cycles each of 10 seconds at 98°C, 30 seconds at 55°C and 1 minute at 72°C followed by final elongation for 7 minutes at 72°C. The final PCR products were undergone partial genomes sequencing with 3730xl DNA sequencer and BigDye Terminator v3.1 Cycle Sequencing Kit (Applied Biosystems, Foster City, CA, USA) using the corresponding primer set as shown in Table 1B.

**Table 1:** SARS-CoV-2 specific primers used for partial sequences of particular region.

**(a) Primers used in Polymerase Chain Reaction**				
	**Stage polarity**	**Primer name**	**Nucleotide position**	**Nucleotide sequence (5’-3’)**
**hCoV-A set**	PCR 1^st^ Sense	SC2-A-1-1	8561–8581	GTTAATAATTGGTTGAAGCAG
	PCR 1^st^ Antisense	SC2-A-1-2	8865–8884	TATCGTGCCAGGCAAACCAG
	PCR 2nd Sense	SC2-A-2-1	8630–8650	TTAATAACACCTGTTCATGTC
	PCR 2nd Antisense	SC2-A-2-2	8834–8854	ACCCACTTCTCTTGTTATGAC
**hCoV-B set**	PCR 1st Sense	SC2-B-1-1	10922–10943	GAATTTACACCTTTTGATGTTG
	PCR 1st Antisense	SC2-B-1-2	11280–11300	GCTTAAAACCAGACAAACTAG
	PCR 2nd Sense	SC2-B-2-1	10957–10977	AGGTGTTACTTTCCAAAGTGC
	PCR 2nd Antisense	SC2-B-2-2	11234–11254	AATACGCATCACCCAACTAGC
**hCoV-C set**	PCR 1st Sense	SC2-C-1-1	14199–14218	AGAGTCACATGTTGACACTG
	PCR 1st Antisense	SC2-C-1-2	14524–14545	TAAAACTAAGTCTAGAGCTATG
	PCR 2nd Sense	SC2-C-2-1	14297–14318	ATTTTAAATATTGGGATCAGAC
	PCR 2nd Antisense	SC2-C-2-2	14500–14521	AGTTTACATCCTGATTATGTAC
**hCoV-D set**	PCR 1st Sense	SC2-D-1-1	23249–23270	CAACAATTTGGCAGAGACATTG
	PCR 1st Antisense	SC2-D-1-2	23496–23516	CTATTAAACAGCCTGCACGTG
	PCR 2nd Sense	SC2-D-2-1	23278–23297	TACTGATGCTGTCCGTGATC
	PCR 2nd Antisense	SC2-D-2-2	23453–23472	GAATAAACACGCCAAGTAGG
**hCoV-E set**	PCR 1st Sense	SC2-E-1-1	25359–25380	AAGGAGTCAAATTACATTACAC
	PCR 1st Antisense	SC2-E-1-2	25658–25678	GCAAAAGGTGTGAGTAAACTG
	PCR 2nd Sense	SC2-E-2-1	25394–25416	TGGATTTGTTTATGAGAATCTTC
	PCR 2nd Antisense	SC2-E-2-2	25634–25654	CAAACAACAACAGCAAGTTGC
**hCoV-F set**	PCR 1st Sense	SC2-F-1-1	25878–25898	TGAACATGACTACCAGATTGG
	PCR 1st Antisense	SC2-F-1-2	26297–26317	AGTACGCACACAATCGAAGCG
	PCR 1st and 2nd Sense	SC2-F-2-1	25919–25939	AATCTGGAGTAAAAGACTGTG
	PCR 1st and 2nd Antisense	SC2-F-2-2	26259–26279	GTAACTAGCAAGAATACCACG
	PCR 2nd Sense	SC2-F-3-1	25948–25968	CACAGTTACTTCACTTCAGAC
	PCR 2nd Antisense	SC2-F-3-2	26216–26237	CTATTAACTATTAACGTACCTG
**hCoV-G set**	PCR 1st Sense	SC2-G-1-1	28612–28631	ATGGGTTGCAACTGAGGGAG
	PCR 1st Antisense	SC2-G-1-2	28903–28923	GACATTTTGCTCTCAAGCTGG
	PCR 2nd Sense	SC2-G-2-1	28709–28729	GGAACAACATTGCCAAAAGGC
	PCR 2nd Antisense	SC2-G-2-2	28874–28893	TCAAGCAGCAGCAAAGCAAG
hCoV-	PCR 1st Sense	ORF8-1-1	27645–27667	TAAACTGTTCATCAGACAAGAGG
**ORF8 set**	PCR 1st Antisense	SC2-H-1-2	28314–28332	GGTCCACCAAACGTAATGC
	PCR 1st and 2nd Sense	ORF8-2-1	27706–27727	GCGGCAATAGTGTTTATAACAC
	PCR 2nd Sense	ORF8-3-1	27779–27800	CTTCTATTTGTGCTTTTTAGCC
	PCR 2nd Antisense	ORF8-2-2	28276–28297	ATTTTGGGGTCCATTATCAGAC
**(b) Primers used in region-specific partial sequencing**				
	**Primer name**	**Nucleotide position**	**Nucleotide sequence (5’-3’)**	**Target Nucleotide position**
**hCoV-A set**	SC2-A-2-1	8630–8650	TTAATAACACCTGTTCATGTC	nt8782
	SC2-A-2-2	8834–8854	ACCCACTTCTCTTGTTATGAC	
**hCoV-B set**	SC2-B-2-1	10957–10977	AGGTGTTACTTTCCAAAGTGC	nt11083
	SC2-B-2-2	11234–11254	AATACGCATCACCCAACTAGC	
**hCoV-C set**	SC2-C-2-1	14297–14318	ATTTTAAATATTGGGATCAGAC	nt14408
	SC2-C-2-2	14500–14521	AGTTTACATCCTGATTATGTAC	
**hCoV-D set**	SC2-D-2-1	23278–23297	TACTGATGCTGTCCGTGATC	nt23403
	SC2-D-2-2	23453–23472	GAATAAACACGCCAAGTAGG	
**hCoV-E set**	SC2-E-2-1	25394–25416	TGGATTTGTTTATGAGAATCTTC	nt25563
	SC2-E-2-2	25634–25654	CAAACAACAACAGCAAGTTGC	
**hCoV-F set**	SC2-F-3-1	25948–25968	CACAGTTACTTCACTTCAGAC	nt26144
	SC2-F-3-2	26216–26237	CTATTAACTATTAACGTACCTG	
**hCoV-G set**	SC2-G-2-1	28709–28729	GGAACAACATTGCCAAAAGGC	nt28881
	SC2-G-2-2	28874–28893	TCAAGCAGCAGCAAAGCAAG	
**hCoV-ORF8 set**	ORF8-3-1	27779–27800	CTTCTATTTGTGCTTTTTAGCC	nt27906-nt27907

**Fig 1:**
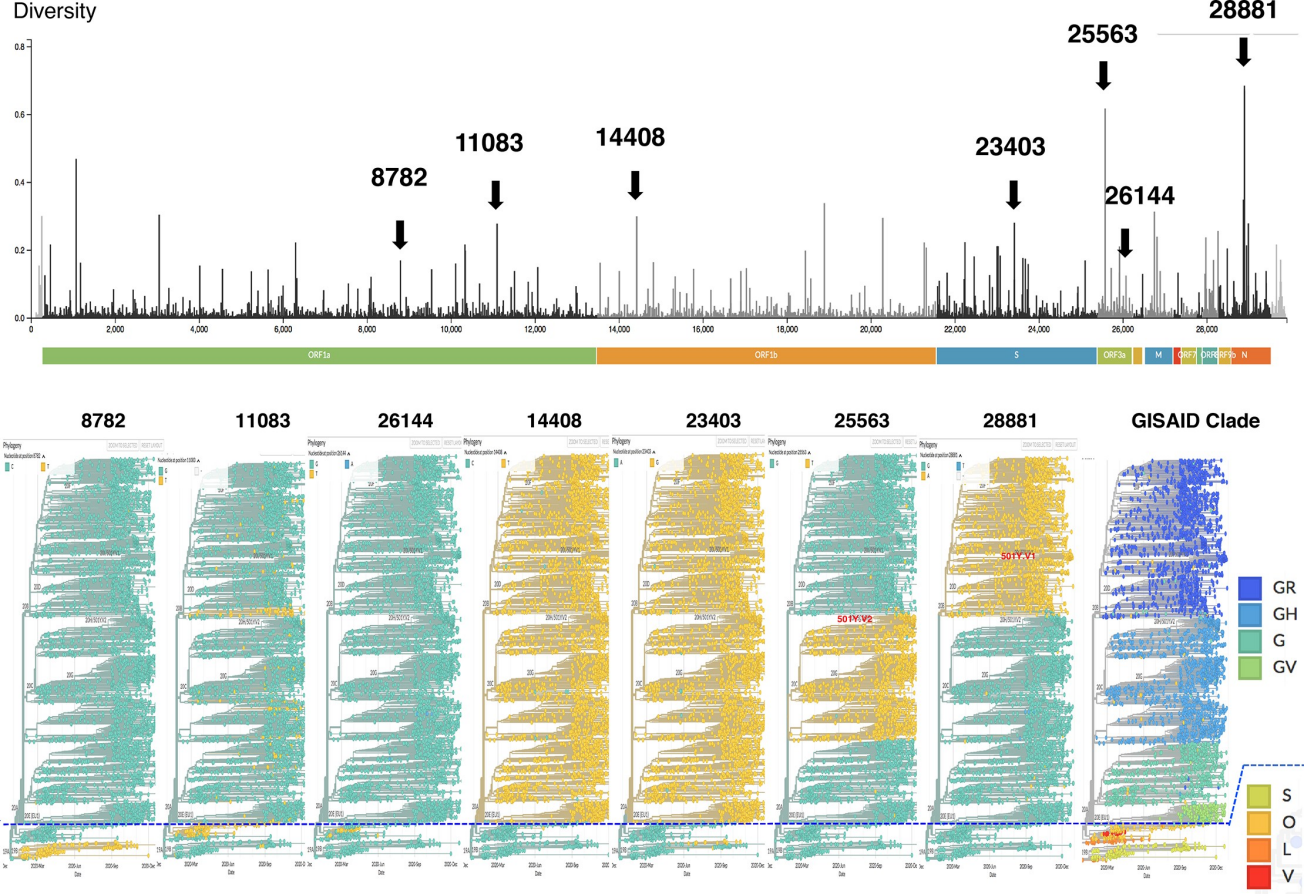
Distinctive variation of nucleotide in specific position for each GISAID clade. This figure shows the spectrum of different nucleotide at their specific position which can be used to classify the GISAID clade. Each color represents the aforementioned base (A: Adenine, G: Guanine, T: Thymine) Asian clade includes S, V, O L and European clade includes G, GH, GR and GV.

In order to differentiate between GISAID Asia and European clade, we examined nt23403 where Adenine (A) for European clade and Guanine (G) for Asian clade. For Asian clade, we subsequently examined at nt8782 (Thymine (T) for Asian clade S, nt11083 (T for Asian clade V or O), nt26144 (T for Asian clade V) and none of them at nt8782, nt11083 and nt26144 as Asian clade O. For European clade, we subsequently examined at nt25563 (T for European clade GH), at nt28881 (A for European clade GR) and the rest as European clade G. (Fig 1)

### Full-length genome sequencing

Full-length genome sequence was acquired by next-generation sequencing (NGS). First, template RNA was translated into complementary DNA (cDNA) using the SuperScript IV First-Strand Synthesis System (Thermo Fisher Scientific). The cDNA was then processed by multiplex polymerase chain reaction (PCR) using the Q5 HoT START DNA polymerase kit and primer pool stocks 1 and 2. The PCR products in the pool 1 and 2 reactions for the same sample were mixed and purified with AmpureXP (1x volume), and full genome sequencing was performed on an Illumina MiSeq System using the QIAseq FX DNA Library Kit. The quality of the sequencing library was assessed using the Quant-iT PicoGreen dsDNA Assay Kit (Thermo Fisher Scientific). The expected amplicon size was 250 bp. The resultant fragments were clustered using MiSeq Reagent Kit v3 and PhiX Control Kit v3, and sequences were analyzed using the MiSeq Control Software (MCS) v2.6.2.1, Real-Time Analysis (RTA) v1.18.54, and bc12fastaq v2.17.

### Phylogenetic tree analysis

A total of 651 SARS-CoV-2 whole-genome sequences were collected from the GenBank (http://www.ncbi.nlm.gov/genbank/) and Global Initiative on Sharing All Influenza Data (GISAID: http://www.gisaid.org). Sequences duplicated between sources were checked and excluded manually. Sequences with many ambiguous sites (N) were removed before analysis. Using 52 SARS-CoV-2 whole-genome sequences from around the world as controls, 522 SARS-CoV-2 whole-genome sequences from throughout Japan and eight full-length genomes isolated in this study were subjected to evolutionary analysis by the neighbor-joining method using Molecular Evolutionary Genetics Analysis (MEGA) version 7.0 [10].

### Examination of molecular characterization and mutation of SARS-CoV-2 strains from Hiroshima

Using the reference strains (MN908947) from GenBank, eight SARS-CoV-2 whole-genome sequences obtained in this study were aligned using MUSCLE multiple sequence alignment algorithms in MEGA v7.0, and then transformed into amino acid sequences. In all whole-genome sequences, the mutations were compared between the reference strain and SARS-CoV-2 in each fragment of *ORF1a*, *ORF1b*, *S*, *ORF3a*, *E*, *M*, *ORF6*, *ORF7a*, *ORF8*, *N*, and *ORF10*. The mutation rate was calculated based according to the sample collection date, and then transformed into units of mutations per year and per 1kb.

### Screening, identification and relativeness of ORF8 deletion in our study with the strains in whole Japan retrieved from GISAID

We used the aforementioned similar partial sequencing method to identify and screen the remaining 60 SARS-CoV-2 samples of Hiroshima using the primer hCoV-ORF8 set (Table 1) targeting the ORF8 region of all samples. Thereafter, we searched through GISAID for the similar ORF8 deletion mutants in the whole Japan and then investigate the molecular pattern between our study and the reported strains.

### Ethic consideration

This study was approved by the Ethics Committee of Hiroshima University (E2124). All participants provided the written informed consent at the beginning of the study. All procedures strictly adhered to the guidelines and the Declaration of Helsinki.

## Results

### Quantitative measurement of SARS-CoV-2

**Fig 2:**
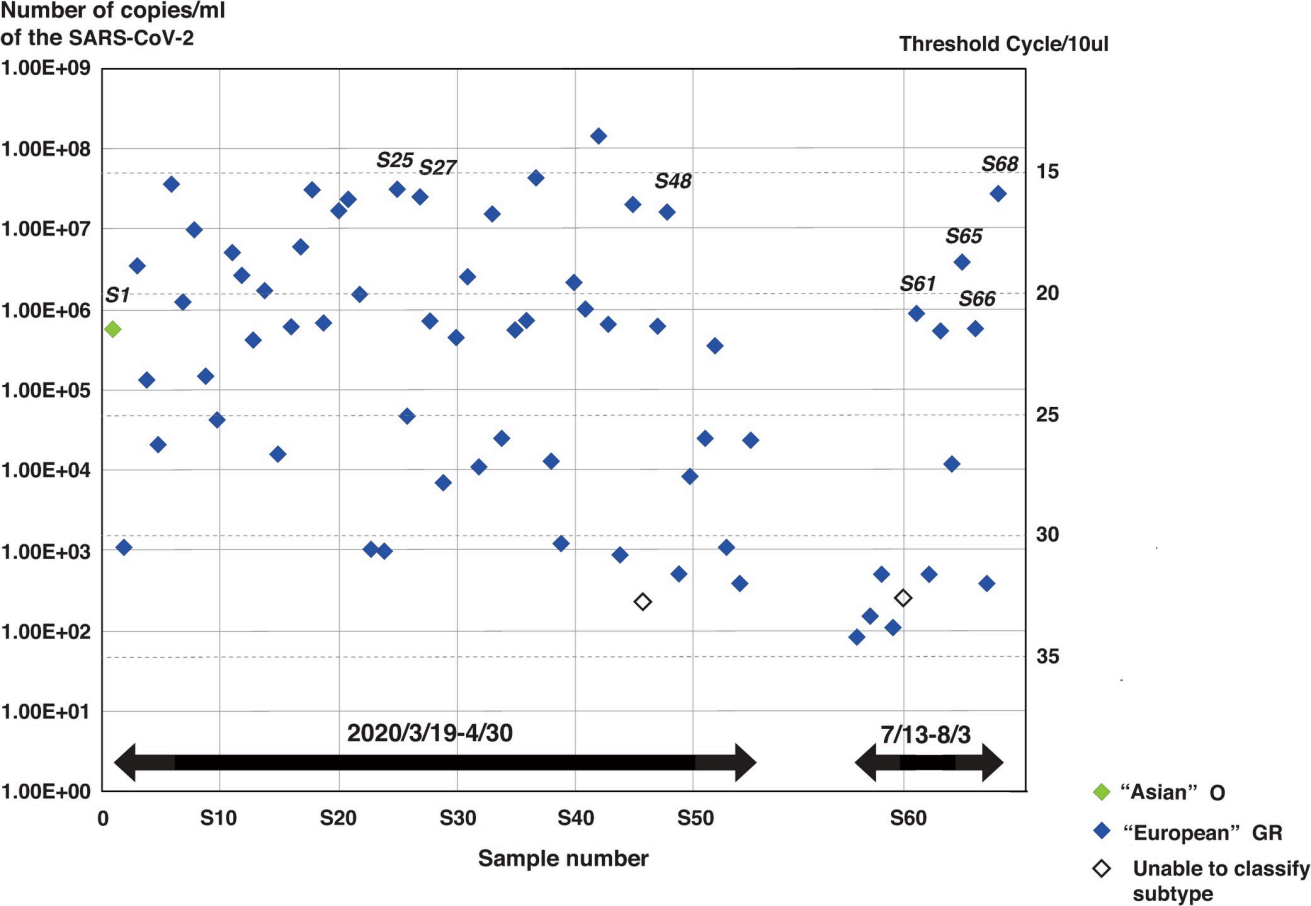
Variation in viral titers of SARA-CoV-2 in Hiroshima. Quantitative measurement of SARS-CoV-2 in 68 samples classified by GISAID clades. Two could not be classified as GISAID clade subtype G, GH, or GR; one was Asian strain O (green dot); and 65 were European strain GR (shown by blue dots). Titers were plotted against cycle threshold for each sample.

Using RT-qPCR, the Asian type yielded a viral load of 5.45 × 10^5^ copies/ml. European strains from the first wave had viral loads of 2.18 × 102–1.42 × 10^8^ copies/ml, and those from the second wave had viral loads of 7.85x10^1^–9.16 × 10^6^ copies/ml (Fig 2).

### GISAID clade (genotype) of SARS-CoV-2 in Hiroshima

Sixty-six (66) of 68 samples were available for partial sequencing to classify GISAID clade (97.1%), whereas two samples were undetectable (2.9%). Both of those undetectable samples had low viral loads, 2.42 × 10^2^ and 2.18 × 10^2^ copies/ml, respectively (Fig 2). Among two samples collected 1 week apart in March, the first isolate was Asian type O and the latter was European type GR. Thereafter, all remaining samples were collected between April 2020 and August 2020, covering the first and second waves; all were European type GR.

### Phylogenetic tree analysis

**Fig 3:**
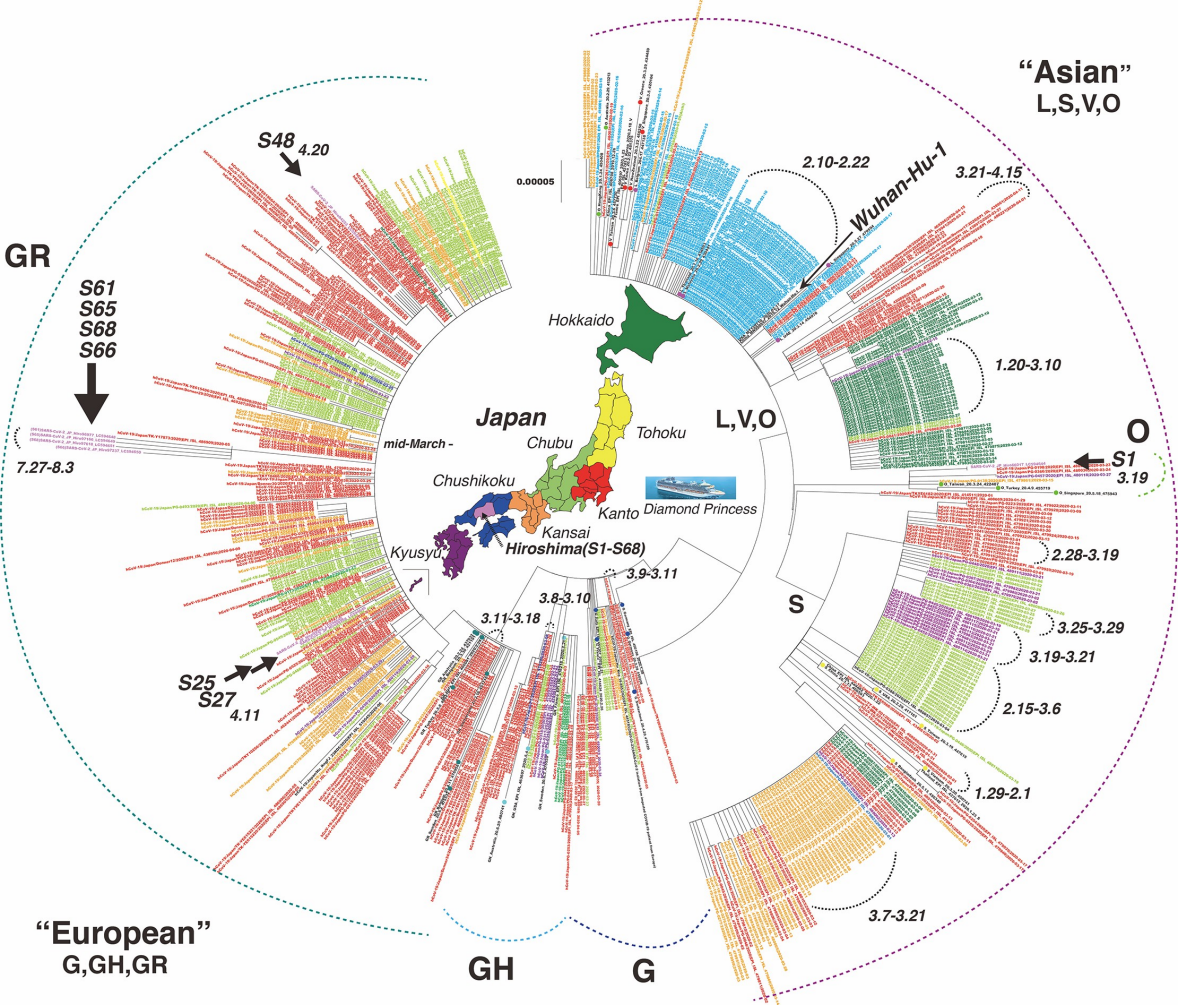
Phylogenetic tree of SARS-CoV-2 in Japan, including eight SARS-CoV-2 isolates from Hiroshima. This phylogenetic tree includes 52 control strains from around the world, 522 strains from Japan retrieved from GISAID and GenBank, and 8 SARS-CoV-2 strains isolated in this study. Controls are shown as colored dots. The strains from Japan are indicated by colored letters corresponding to the colors shown in the map of Japan, in which the country is divided into seven main regions. The sky-blue color represents the strains from the Diamond Princess cruise. Our eight SARS-CoV-2 strains are shown in purple and are also indicated by sample number. The tree was performed by the neighbor-joining method using MEGA7. There were total of 29038 positions in the final dataset.

Using all isolates confined to Japan and controls retrieved from GISAID and GenBank, a phylogenetic tree was constructed and categorized by region of Japan: from North to South, Hokkaido, Tohoku, Kanto, Chubu, Kansai, Chushikoku (originally Chugoku and Shikoku separately, but combined in our study), and Kyushu (Fig 3). In Japan, the Asian type of SARS-CoV-2 spread from January to April 2020, and many small clusters of cases occurred transiently in the Chubu, Kansai, and Hokkaido regions, as well as the on the Diamond Princess Cruise. Various types of Asian clades were circulating in these areas. Later, starting in the first week of March, clusters of European types G and GH appeared. Since mid-March, a number of European GR clusters have been observed simultaneously across the country; subsequently, this strain became the predominant type in Japan. In Hiroshima, analyses of eight full genomes revealed that one (S1: SARS-CoV-2_JP_Hiro66017_LC584644) isolate belonged to Asian type O; the rest were European type GR, in which S25 (SARS-CoV-2_JP_Hiro99262_LC594645), S27 (SARS-CoV-2_JP_Hiro99268_LC594646), and S48 (SARS-CoV-2_JP_Hiro94253_LC594647) were circulating in the first wave and S61 (SARS-CoV-2_JP_Hiro96977_LC594648), S65 (SARS-CoV-2_JP_Hiro97166_LC594649), S66 (SARS-CoV-2_JP_Hiro97237_LC594650), and S68 (SARS-CoV-2_JP_Hiro97618_LC594651) were circulating in the second wave (Fig 3). The second wave GR strains appeared independently with 11–15-base mutations.

### Mutations and the subsequent amino acid changes in SARS-CoV-2 strains in Hiroshima

**Fig 4:**
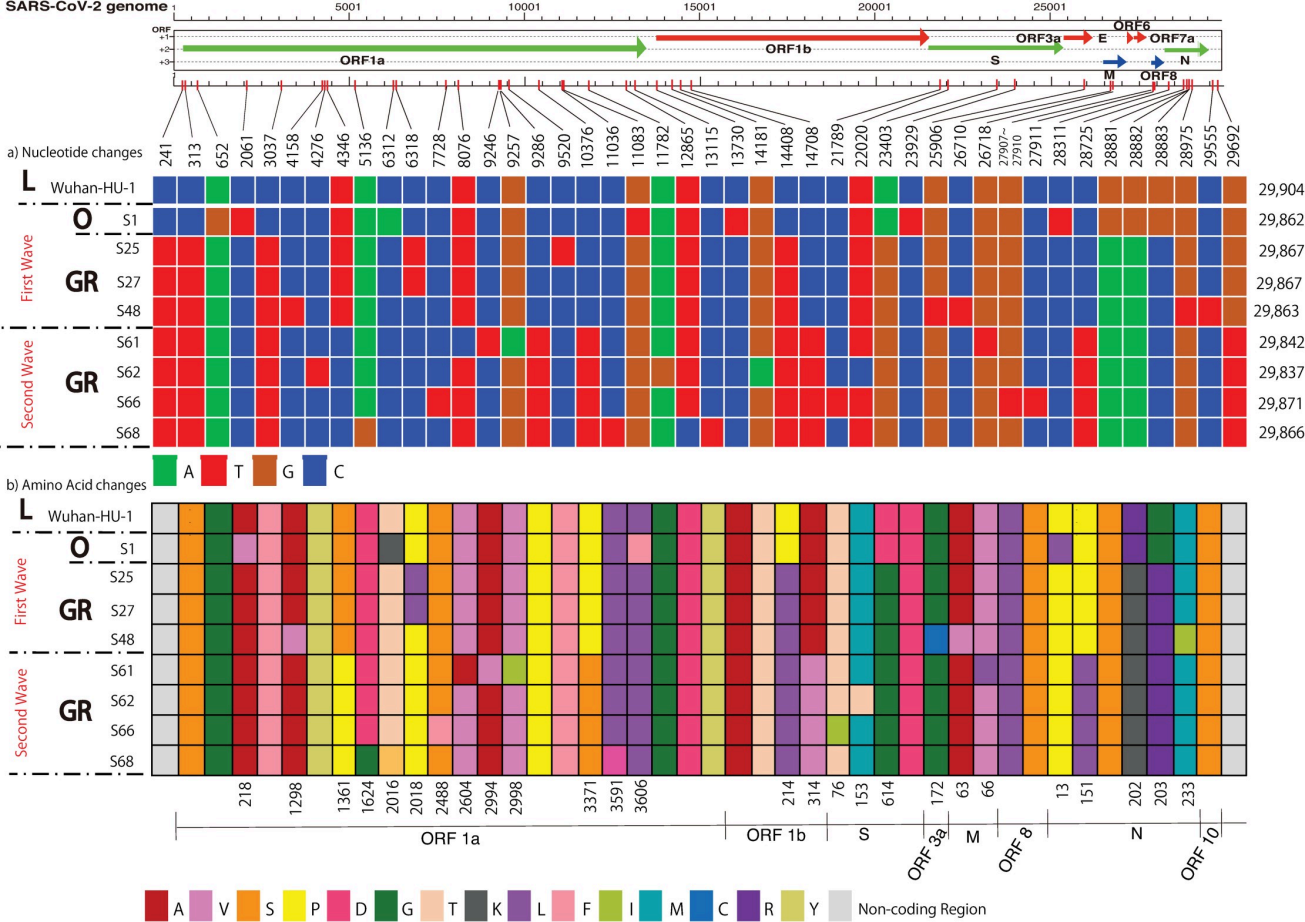
Nucleotide mutations and amino acid replacements in the whole genomes of SARS-CoV-2 isolates from Hiroshima. This figure shows a) nucleotide mutations and b) resultant amino acid changes identified in eight SARS-CoV-2 isolates from Hiroshima. Colors represent nucleotides or amino acids, according to the legend below each panel. Genomic regions are indicated by the whole-genome model at the top, and point mutations are indicated by the color changes.

Relative to reference strains (MN908947), seven different types of mutation were found at 44 different sites among the eight isolates of SARS-CoV-2; 59.1% (26/44) were nonsynonymous mutations, and the rest (40.9%) were synonymous mutations. Among these 44 sites, *ORF1a* and *1b* contained 27 changes (61.4%), nucleocapsid (*N*) contained five changes (11.4%), and spike (*S*) had four changes (9.1%) (Fig 4).

Seven nucleotide mutations were found in the Asian O strain, amongst which five were located in *ORF1a*; one each was in *S* or *N*. All European type GR strains in the first wave had similar mutations at eight different sites: one in a non-coding region (C241T), two in *ORF1a* (C313T, C3037T), one in *ORF1b* (C14408T), one in *S* (A23403G), and three in *N* (G28881A, G28882A, G28883C); half were nonsynonymous. Additionally, several discrete mutations were also found at various sites in particular strains, as shown in Fig 3. Overall, there were 32 mutations at 15 sites in European type strains of the first wave: 50% (16/32) were in *ORF1ab*, 31.3% (10/32) in *N*, 9.4% (3/32) in *S*, and 3.1% (1/32) each in *ORF3a*, *ORF10*, and *M* (Fig 4).

The aforementioned eight similar mutations also occurred in all European strains of the second wave. Additionally, another similar pattern of mutations was shared among these strains at six different sites: three (T4346C, C9286T, C10376T), one (C14708T), one (C28725T), and one (G29692T) in *ORF1a*, *ORF1b*, *N*, and non-coding regions, respectively; four of them were nonsynonymous. Moreover, 16 discrete nucleotide changes were also found at various sites in particular strains (Fig 3). Thus, there were 72 mutations at 30 sites in European strains of the second wave: 59.7% (43/72) were in *ORF1ab*, 22.2% (16/72) in *N*, 8.3% (6/72) in *S*, 5.5% (4/72) in a non-specific region, 2.8% (2/72) in *ORF8*, and 1.4% (1/72) in *M* (Fig 3). Notably, all seven European strains shared a triple mutation at G28881A, G28882A, and G28883C in the *N* gene. The estimated mutation rate was 38.4 nucleotides per year and 1.16–1.87 × 10^−3^ base substitutions per site per year. The number of mutations within the first wave ranged from one to seven changes, but in the second wave, it was eight changes.

Four amino acid substitutions were found in Asian strain O: three (218: Arginine to Glycine, 2016: Threonine to Lysine, 3606: Leucine to Phenylalanine) were in ORF1a and one (13: Proline to Leucine) was in N. Four similar patterns of amino acid substitutions were found in all European strains from both the first and second waves: ORF1b had one (214: Proline to Leucine), spike had one (614: Aspartic acid to Glycine), and nucleocapsid had two (2020: Arginine to Lysine and 203: Glycine to Arginine). Notably, all four European strains from the second wave shared four similar patterns of amino acid substitutions (1361: Serine to Proline and 3371: Proline to Serine in ORF1a, 314: Alanine to Valine in ORF1b, and 151: Proline to Lysine in nucleocapsid). Moreover, the rest of the amino acid substitutions were individually distributed at various sites in particular European strains. The majority of amino acid substitutions were found in non-structural proteins (ORF1a and 1b), accounting for 57.7% of total substitutions. Among structural proteins, N (15.4%) and S (11.5%) exhibited the most frequent amino acid substitutions sites in all European strains, and other substitutions were also found in ORF3a and M (Fig 4).

### The D614G variants of European strain GR

The D614G amino acid change was found in the spike region of European strain GR, and this amino acid change was accompanied by the silent mutation of C241T in a non-coding region, C313T and C3037T in *ORF1a*, the nonsynonymous mutation at C14408T (P214L) in *ORF1b*, and triple mutation of G28881A, G28882A, and G28883C resulting in amino acid changes (R202K and G203R) in N. Notably, the spike region contained four nucleotide mutations resulting in three amino acid changes, as shown in Fig 3. Additionally, the D614G variant of S65 had the T22020C mutation in the same region, and S66 had the C21789T mutation in the spike region; both of these mutations resulted in amino acid changes (153: Methionine to Threonine and 76: Threonine to Isoleucine) (Fig 4).

### Single-base insertion in SARS-CoV-2_JP_Hiro97327_LC594650

**Fig 5:**
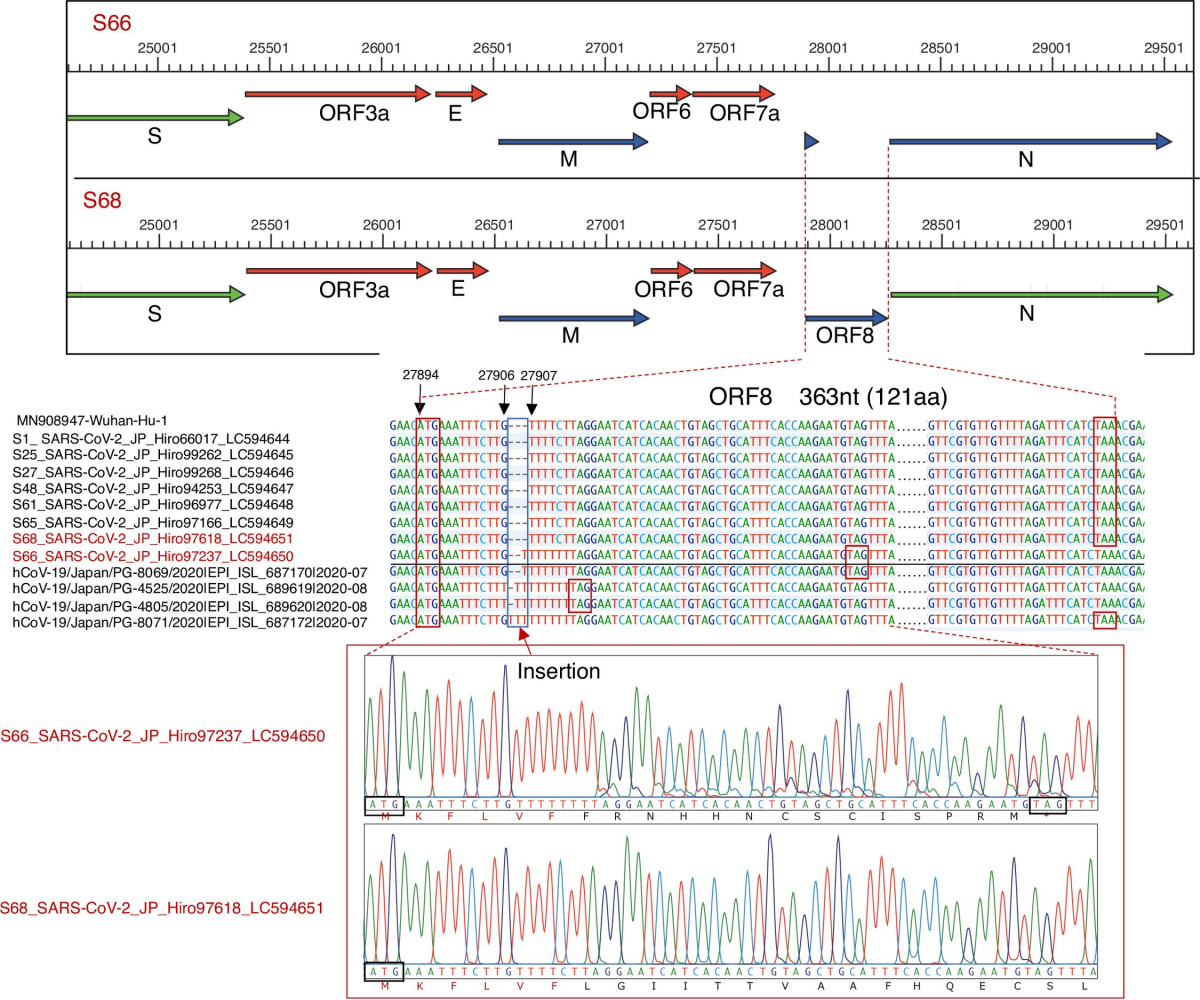
Single-nucleotide insertions in open reading frame (ORF8) resulting in frameshift and ORF8 deletion. The framed box at the top compares ORF8 between wild-type S68 and mutant variant S66, using whole-genome sequences obtained by next-generation sequences (NGS). Disappearance of ORF8 was observed in the sample. Additionally, 4 SARS-CoV-2 strains of Japan having the similar mutation pattern retrieved from GISAID were also included in this figure. This small peak results from mixed cloning of SARS-CoV-2 mutants with single and double Thymine (T) nucleotide insertion between nt27906 and nt27907 whereas the large dominant wave represent the dominant mutants single Thymine insertion and small peak represents the recessive mutants with double Thymine insertion.

A single-nucleotide insertion was found in sample S66, collected during the second wave of the outbreak in Hiroshima. The nucleotide insertion was assumed to have occurred between nt27906 and nt27907 of ORF8, which contains 363 nucleotides (121 amino acid). This single-nucleotide insertion caused a frameshift and consequently the disappearance of ORF8, as shown in Fig 4. Instead of the usual length of 121 amino acids in the wild type, ORF8 of this mutant variant was shortened to 20 amino acids. As a result, only initial six amino acids were the same as the wild type, and the rest differed due to the frameshift. (Fig 5) Among 9,844 SARS-CoV-2 strains of the whole Japan submitted at GISAID as of January 12, 2021, we found only 4 mutant strains having similar nucleotide insertion (one has single base insertion, two have double base insertion and one has triple base insertion) with subsequent frameshift and early appearance of stop codon as shown in Fig 5. As per clinical profile, this patient, 60 years old man, infected with ORF8 deletion mutant, had mild symptoms only and cured after 13 days of hospitalization.

## Discussion

This study revealed the molecular characteristics and mutation pattern of SARS-CoV-2 strains circulating in Hiroshima from the day of first confirmed case to August 3, 2020. At that time, the total number of cases in Hiroshima Prefecture was 366 while the whole country has reported 40,244 new confirmed cases. It means Hiroshima had 0.9% of total cases in Japan at that time. Among them, 68 samples were provided from Hiroshima Prefecture Research Institute after being used by government laboratory and we performed real-time PCR for quantitative measurement of virus and region specific-partial sequencing for GISAID classification as the first step. Then, we randomly selected 8 samples to undergo NGS full genome sequencing. As of January 14, 2021, 9,885 strains (3.3%) out of 298,172 confirmed cases in Japan are able to register at GISAID. Our study could report 8 (2.2%) out of 366 almost the same as the whole Japan. Interestingly, among 68 samples, only one strain collected during the first wave was of the Asian type; the remaining strains from both the first and second wave of the outbreak were GISAID clade GR. Our phylogenetic tree analysis explained the evolution of SARS-CoV-2 in Japan, although the origin and cascade of transmission were difficult to determine. From January through April 2020, a large number of case clusters occurred on the Diamond Princess Cruise and in the Hokkaido, Kanto, Kansai, Chubu, and Kyushu regions; these clusters shared various Asian strains (L, S, V, and O) and were confined to particular areas. In the second week of March, the European strains G, GH, and GR entered Japan and were mainly found in the Kanto region. After mid-March, European strain GR was predominant throughout the whole of Japan. The full-length genomes obtained in our study revealed that all seven European strains were genotype GR (specifically, D614G variants). Japan had an urgent response to the outbreak, and the alert level was upgraded in late March. Our study showed that the Asian strains were less likely to cause further outbreaks after the strict measures against coronavirus, and were no longer reported in Japan. This implies Asian strain had a low level of replication and was less transmissible than the European strains. Therefore, the strict public health measures could interrupt the chain of transmission. By contrast, the European strains have higher infectivity, virulence, and replication rate than the Asian type, especially the D614G mutant variants [11]. Our study also indicated that European strains had higher viral loads than Asian strain, suggesting higher replication efficiency, and all 7 European strains of type GR had high mutation rates. Under strict public health measures, new cases decreased temporarily, but evolution of SARS-CoV-2 occurred during this period. Subsequently, new episodes of outbreak was occurred by newly emerged mutant variants with differences in virulence, infectivity, replication, and transmissibility.

Among all proteins in SARS-CoV-2 strains in Hiroshima, both Asian and European strains had amino acid substitutions as a result of specific single-base mutations. Because SARS-CoV-2 is an RNA virus, the mutation rate is high, leading to continued evolution over time that might affect replication, infectivity, transmissibility, virulence, and immunogenicity [12]. Our study also showed that the mutation rate between the first and second waves was 34.9–40.6 nucleotides per year, corresponding to 1.17–1.36 × 10^−3^ base substitutions per site per year. This mutation rate is lower than that of hepatitis C virus [13]. A recent study reported that the mutation rate of SARS-CoV-2 in variable sites was 3.5 × 10^−3^ changes per site per year, higher than the value we measured [14]. Interestingly, all European strains shared similar patterns of mutation at specific sites, and the accompanied amino acid substitutions were mostly found in polyprotein OFR1ab rather than in structural proteins. Md Rafiqul Islam *et al*. also reported that most nucleotide mutations occurred in the polyprotein, with ORF1a being the most frequently mutated region [15]. Our findings are consistent with previous reports. Mutations in ORF1a and 1b might influence the replication efficiency of the virus because they are essential for the main protease (Mpro) and replicase involved in processing of polyproteins and control of viral replication [16, 17].

Notably, we found a mutation in the nucleocapsid protein in all seven GR strains from Hiroshima: a triple mutation (G28881A, G28882A, and G2888C) resulting in an amino acid change. Moreover, four GR strains from the second wave had the same mutation at C28725T. The nucleocapsid proteins play important roles in RNA replication, transcription, and genome packaging [18, 19]. The high frequency of mutation in the genomes, particularly in the structural proteins, promotes the emergence of new strains of the virus with differences in morphological structure, virulence, infectivity capacity, and replication efficiency. This phenomenon complicates the development of vaccine candidates and drugs. Additionally, we identified a predominant type of D614G variants with the concomitant silent mutations C241T, C313T, and C3037T in ORF1a; the P214L amino acid substitution in S; and double mutation of R202K and G203R in N. A recent study suggested that D614G variants became the predominant type circulating worldwide; these strains account for 78% of total distribution as of May 2020 and are associated with increased infectivity [20] and a high case-fatality rate [11]. In light of the alterations in infectivity and transmissibility of D614G variants, our findings provide important insights into clinical management of SARS-CoV-2, as well as how we might modify preventive and control strategies in the future. In addition to the D614G mutation, the spike region contained two different types of mutations, M153T in S65 and T76I in S66. The spike proteins are key surface glycoproteins that play essential roles in viral entry and interact with host cell receptors [21, 22]. Hence, a high mutation in the spike region might interfere with the development of vaccines or antibody-based therapeutic agents.

Another noteworthy finding is the detection of a single-nucleotide insertion between nt27906 and 27907, resulting in deletion of the ORF8 protein. A SARS-CoV-2 variant with a 382-nucleotide deletion was reported in Singapore, Bangladesh, Australia, and Spain [23–25]. This type of deletion removes an ORF8 regulatory sequence, eliminating ORF8 transcription; the variant is associated with milder infectivity and lower levels of cytokine release during the acute phase [25]. Although the role of ORF8 of SARS-CoV-2 remains unclear, one study suggested that it disrupts antigen presentation and decreases the recognition and elimination of virus-infected cells by cytotoxic T lymphocytes (CTLs) [26]. In our study, we suggested that single base insertion in ORF8 causes the subsequent amino acid frameshift and early appearance of the stop codon, with concomitant disappearance of ORF8. We found only one case among eight full-length genome and 60 partial genomes. Additionally, only 4 similar mutants strains were reported among 9844 strains of the whole Japan submitted at GISAID. Therefore, we suggested that the transmission of ORF8 deletion mutants was temporarily occurred during the second wave showing the less longevity, less viral replication, less transmission and less infectivity. The finding is consistent with the recently published report [24].

Our study had some limitations. Because of insufficient sample volume, it is impossible for all samples to do full-genomes sequencing. However, we decreased selection bias by random blinded selection of eight samples for whole-genome sequences analysis but the random error cannot be excluded. Although the sample size is small, this is the very first report about the full-genomes of SARS-CoV-2 strain in Hiroshima as well as the Japan, so that it is very precious data and findings which can be used as the baseline for the further molecular surveillance as well as the clue for development of vaccine, immunotherapy and diagnostic tool. Moreover, it is necessary to conduct the large study to correlate between mutation result and their clinical outcomes in patients, infectivity, virulence, or viral factors in the future. As our study focused on phylogeny, molecular characteristics and their mutation patterns, we did not examine detailed morphological changes or alterations of biological functions caused by the mutations we identified.

In conclusion, our findings revealed that the recently emerged strain of SARS-CoV-2 in Hiroshima was European clade GR. The transmission cascade and high mutation rate might be associated with alteration of infectivity and transmissibility of SARS-CoV-2 strains in this region. The predominant D614G variants and a new form of ORF8 deletion also provide the clues for the role of viral factor in local outbreak of SARS-CoV-2 in Hiroshima. Understanding the molecular characteristics and mutation patterns over time would be useful in clinical settings, in design of public health measures, and for development of vaccines and therapeutic drugs. Further study and molecular surveillance of circulating strains are required. In addition, it will be necessary to optimize clinical management of novel coronavirus infection.
